# The importance of considering the duration of extreme temperatures when investigating responses to climate change

**DOI:** 10.1111/gcb.16381

**Published:** 2022-08-19

**Authors:** Teija Isotalo, Lilla Rotenbiller, Ulrika Candolin

**Affiliations:** ^1^ Organismal and Evolutionary Biology University of Helsinki Helsinki Finland

**Keywords:** behaviour, climate change, courtship, global warming, heatwave, phenotypic plasticity, reproduction, spawning

## Abstract

The frequency and duration of heatwaves are increasing because of human activities. To cope with the changes, species with longer generation times may have to rely on plastic responses. The probability that their responses are adaptive is higher if the species have experienced temperature fluctuations also in their evolutionary past. However, experimental studies investigating responses to heatwaves often use exposure times that are significantly shorter than recent heatwaves. We show that this can lead to faulty conclusions and that the duration of higher temperature has to be considered in experimental designs. We recorded the response of threespine stickleback to prolonged duration of higher temperature during the breeding season, using a population that has experienced large fluctuations in temperature in its past and, hence, is expected to endure temperature changes well. We found males to adaptively adjust their reproductive behaviours to short periods of higher temperature, but not to longer periods that extended across two breeding cycles. Males initially increased their reproductive activities—nest building, courtship and parental care—which ensured high reproductive success during the first breeding cycle, but decreased their reproductive activities during the second breeding cycle when exposed to sustained high temperature. This reduced their courtship success and resulted in fewer offspring. Thus, a species expected to cope well with higher temperature suffers fitness reductions when the duration of high temperature is prolonged. The results stress the importance of considering the duration of extreme environmental conditions when investigating the impact that human activities have on species. Responses to short‐term exposures cannot be extrapolated to assess responses to longer periods of extreme conditions.

## Graphical abstract

The frequency and duration of heatwaves are increasing because of human activities. We show that stickleback males are able to adaptively adjust their reproductive behaviours to short‐term increases in temperature, but not to longer‐term periods that extend across two breeding cycles. This highlights the importance of considering the duration of extreme environmental conditions when investigating the impact of human activities on species

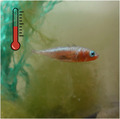

## 1 INTRODUCTION

Climate change is restructuring species communities across the globe (Antao et al., 2020). Assessing the mechanisms behind the changes and the vulnerability of individual species to the changes are important endeavours in our increasingly disturbed world. The knowledge is needed to develop strategies to mitigate large‐scale changes to ecosystems, which otherwise could threaten their functioning and services (McLean et al., 2016; Urban et al., 2016). However, most experimental studies on the effects of climate change on species rely on short‐term exposures to higher temperature. These brief exposures may not reflect the changes occurring in nature or expected to occur as climate change escalates (Noer et al., 2022). Thus, the degree to which existing results can be extrapolated to predict the vulnerability of species to the intensification of climate change is uncertain.

The vulnerability of a species to climate change depends on its ability to plastically adjust (including migration) and genetically adapt to the changes. Species with long generation times in relation to the rate at which the environment is changing may not be able to adapt through genetic modifications but have to rely on plastic adjustments (Barrett & Hendry, 2012; Chevin, 2013; Hendry et al., 2008; Merila & Hendry, 2014). These adjustments depend on existing reaction norms that have evolved under past environmental conditions (Chevin et al., 2010; Chevin & Hoffmann, 2017; Fox et al., 2019; Sih et al., 2011; Tuomainen & Candolin, 2011). Thus, the probability of adaptive responses is higher when the disturbance extends earlier encountered conditions, such as a gradual rise in temperature, than when it creates novel conditions (Candolin & Jensen, 2021; Sih et al., 2011). Nevertheless, various factors may still reduce the adaptiveness of the responses, such as the temporal pattern of the environmental change. In particular, prolonged periods of high temperature can pose a challenge to species that have only encountered shorter spells of higher temperature in their past (Angiletta, 2009; Roman‐Palacios & Wiens, 2020; Stillman, 2019). For instance, while individuals may cope well with brief heatwaves through reduced activity, the strategy may become maladaptive during extended heatwaves, as it may decrease foraging rate or the likelihood of finding partners (Candolin, 2019; Dell et al., 2014).

Temperature fluctuations are particularly troublesome for many ectothermic species, as their metabolism depends on the temperature of the external environment (Abram et al., 2017). Fluctuations that influence their metabolism can alter behaviours that determine fitness, such as reproductive behaviours (Brandt et al., 2018; Conrad et al., 2017; Macchiano et al., 2019). For instance, a short exposure to higher temperature alters courtship displays in the wolf spider *Schizocosa floridana* (Rosenthal & Elias, 2019) and in the field cricket *Gryllus integer* (Hedrick et al., 2002). However, the impact that prolonged duration of higher temperature has on reproductive behaviours is poorly known, as is the ultimate impact that the responses have on reproductive success (Bernal et al., 2020; Spinks et al., 2021; Suryan et al., 2021). Yet, the impact could alter population dynamics given the central role that reproductive behaviours play in determining fitness.

Species that reproduce in shallow coastal waters are particularly exposed to heatwaves, as the water warms up faster than offshore waters (Harvey et al., 2022; Oliver et al., 2021; Smale et al., 2019; Vinagre et al., 2018). The ability of these species to cope with sudden rises in temperature is likely to depend on their past experience of temperature fluctuations and, hence, on the presence of adaptive reaction norms for coping with fluctuations. However, past fluctuations may have been less intense, infrequent or of shorter duration than current ones and, hence, reaction norms based on past selection may not be adaptive under future climate change (La Sorte et al., 2021).

We investigated if a species that reproduces in shallow coastal waters—the threespine stickleback, *Gasterosteus aculeatus*—is able to adjust its reproductive behaviours to increases in water temperature, and if the ability depends on the duration of the higher temperature. Recent research indicates that exposure to higher temperature influences its reproductive success, but whether the impact depends on the duration of the higher temperature is unknown (Fuxjager et al., 2019; Wanzenboeck et al., 2022). We used a population that has experienced temperature changes in the past and which consequently is expected to cope well with brief periods of higher temperature, but whose ability to cope with longer periods is unknown. A recent heatwave in the study area in the summer of 2021 lasted for 50 days, being the warmest ever summer since the recordings started in 1845 (https://en.ilmatieteenlaitos.fi/open‐data), and such conditions are expected to increase in the future (Meier et al., 2019). If a species that has been exposed to periods of higher temperature in the past is not able to adjust to the current increase in the frequency and duration of heatwaves, then species that originate from more stable environments may find it even more challenging. Thus, a failure of stickleback to adjust to prolonged periods of higher temperature could indicate an even stronger effect of climate change on other species.

To investigate the response of the studied threespine stickleback population to prolonged duration of higher temperature, and the impact that the responses have on reproductive success, we exposed males to higher temperature during either one or two breeding cycles. During reproduction, stickleback males build a tunnel‐shaped nest out of algae to which they attract females to spawn using a courtship dance combined with nuptial coloration (Tuomainen & Candolin, 2013). Females leave after spawning and the male alone cares for the eggs in the nest until hatching. A male may complete several breeding cycles during one breeding season, usually two or three cycles (Candolin, 2000a). We recorded effects of the duration of higher temperature on reproductive behaviours—nest building, courtship, and parental care—and the ultimate impact the duration has on their reproductive success, the number of offspring produced. We further assessed if the timing of the temperature increase—early or late during the breeding season—influences the responses. We predicted that the ability of males to maintain high reproductive activity would decline with the duration of higher temperature and that the effect would be more pronounced later in the season when males are in poorer condition (Candolin, 2000a).

## 2 METHODS

### 2.1 Collection and housing

We caught threespine stickleback in early May 2019 before the breeding season from a bay in the outer archipelago of the Northern Baltic Proper (60° N, 23° E) using Plexiglas traps (Candolin & Voigt, 2001) (see map in Supporting Information). Stickleback migrate to the bay from the open sea in the spring to spawn, and leave it at the end of the summer when the breeding season ends. Temperature fluctuates in the bay across days (Granroth‐Wilding & Candolin, 2022) and years (Meier et al., 2022), depending on climate conditions, because of its shallow topography; max depth is about 1.5 m. Thus, the population has experienced fluctuations in water temperature during the breeding season in its evolutionary past. We transported the fish to Tvärminne Zoological station, University of Helsinki, within 20 min. We housed the fish in large flow‐through tanks (salinity 5.5 psu) at a density of 0.25 fish per litre, in an outdoor facility under natural temperature and light conditions. We fed the fish defrosted chironomid larvae once a day.

### 2.2 Experimental design

When males came into reproductive condition, as determined by the development of nuptial colouration, we measured their size (standard length and weight) and transferred them to 10‐L aerated tanks in climate chambers, one male per tank. We exposed them to one of four temperature treatments during two breeding cycles, 18 males per treatment: (1) constant normal temperature of 14°C, NN, (2) constant high temperature of 19°C, HH, (3) switch from high to normal temperature between breeding cycles, HN and (4) switch from normal to high temperature between cycles NH. The constant normal temperature served as the control and the constant high temperature as the long‐term treatment, and the two treatments with switches as short‐term treatments where the higher temperature occurred either early or later during the breeding season. A temperature of 14°C represents average natural temperature in the spawning habitat during the breeding season, as measured in June during 9 years preceding the study (14.1°C ± 0.7, mean ± *SD*, *n* = 9, see Table S1) (see also Granroth‐Wilding and Candolin (2022) and MONICOAST‐project of Tvärminne Zoological Station (www.helsinki.fi/monicoast)). A temperature of 19°C or higher is occasionally attained when air temperature exceeds 25°C for several days. However, during the preceding 9 years, temperatures of 19°C or higher had not lasted more than maximum 5 days (U. Candolin, unpublished data).

The duration of each treatment depended on the time it took a male to complete each breeding cycle, as males usually initiate the second cycle as soon as the first one is completed. Thus, males experiencing a switch in temperature were transferred to the other temperature treatment as soon as they had completed the first cycle. Keeping the duration of the treatments equal across males would have forced us to prevent males from initiating the second breeding cycle—until the slowest male had completed his first cycle—which could have influenced their reproductive behaviour. To change water temperature, we transferred the tanks, containing the males, between the two climate chambers, allowing the water to gradually attain the new temperature, which took about a day. The tanks maintained at constant temperature were exposed to the same disturbance by moving them within the chambers. Males in the four treatments did not differ in body size (length *F*_3,68_ = 0.73, *p* = .54, weight: *F*_3,68_ = 1.73, *p* = .17, see Table S2 for values).

Each male tank contained a nesting dish (Ø 12.5 cm) filled with sand and filamentous algae, *Cladophora glomerata*, for nest construction, and an artificial plant for hiding (Candolin, 1997). LED lights above the tanks were programmed to replicate natural light conditions with dawn and dusk and a light:dark period that changed from 17:7 in May to 18:6 in June. To stimulate nest building, we presented the males with a gravid female, enclosed in a transparent, perforated, plastic jar, three times a day for 10 min. We recorded the time it took each male to build a complete nest, that is, a nest through which he had crept through (Candolin & Salesto, 2006).

### 2.3 Courtship recordings

When a nest was ready, we recorded the courtship behaviour of the male towards three consecutive gravid females, with a 30 min break between presentations. Some females were reused among males, which was considered in the analyses. Each female was enclosed within a perforated, transparent jar and the presentation lasted for 10 min. Females had been kept in the outdoor facility under natural temperature conditions until 1–2 days before experimentation, when they were transferred to an 10‐L aerated tank held at the same temperature as the experimental male tank. We video recorded the courtship behaviour of the male and analysed his behaviour using the software BORIS (Friard & Gamba, 2016): number of leads towards the nest (the male approached the female, sometimes through zigzag movements, and then moves in a straight line towards the nest), number of fanning bouts at the nest entrance (the male fans fresh water into the nest using his pectoral fins), total time spent fanning, and total time spent courting the female.

### 2.4 Spawning and hatching success

After the third courtship recording, we released the female and allowed her to spawn with the male. We recorded the time until spawning occurred. If the female did not spawn within 2 h, we assumed she was not interested in the male and replaced her with a new female. Spawning time was in these cases noted as the maximum time, 120 min. We measured the number of eggs spawned by gently removing all eggs from the nest 2 h after spawning—when the eggs had hardened—and weighing the total clutch to the nearest 0.01 g (Candolin, 2000b). To calculate the number of eggs, we divided the weight of the egg clutch by the mean weight of an egg. The weight of an egg was calculated by measuring the diameter of 10 eggs in the clutch using a camera connected to a cold‐light microscope, and then multiplying mean egg volume with egg density (buoyance), 1.01 g/cm^3^ (Nissling et al., 2017). Females spawn all ovulated eggs at one spawning. The number of eggs received did not differ among treatments (*F*_1,74_ = 1.59, *p* = .21).

After weighing the egg clutch and photographing 10 eggs, which took maximum 10 min and does not influence survival (Candolin, 2000c), we returned the egg clutch to the nest. Males always accepted the eggs and resumed parental care behaviour. We recorded male parental behaviour by video recording the males for 10 min each day. We analysed the behaviours using the software BORIS: number of fanning bouts, total time spent fanning, and total time spent by the nest (which includes gluing, cleaning and prodding into the nest to remove dead or diseased eggs, in addition to fanning).

When the eggs were almost ready to hatch, after 7 days at 14°C and after 5 days at 19°C, based on earlier work (Candolin et al., 2022), we removed the eggs from the nest and transferred them to separate aerated tanks to follow hatching success. We counted the number of fry emerging and calculated hatching success as the percent of eggs received that hatched. The possible presence of unfertilised eggs is unlikely to influence the results, as earlier studies show that average fertilisation success is over 99% in the population (Candolin et al., 2021, 2022).

We allowed the males from the first breeding cycle to complete a second cycle by providing them with fresh material for nest building, and repeated the procedures from the first cycle. Four males did not enter a second cycle; one at normal temperature, and three at high temperature. In the analyses, qualitatively similar results are gained if these males are removed from the analyses or maintained as missing values during the second cycle. Given that we do not know the reason for the males not entering a second breeding cycle, we present the more conservative results with the males removed from the analyses. The total number of eggs received across the two breeding cycles did not differ among treatments (*F*_3,68_ = 0.84, *p* = .48).

We measured the length and weight of the males at the end of the second breeding cycle and calculated changes in body condition as percent weight lost and as change in Fulton's condition factor K (Le Cren, 1951). Qualitatively similar results were gained using the two measures and we present the data for percent weight lost. We fed the males defrosted chironomids during the nest building stage, but not during courtship and parental care, as males do not feed during this period.

### 2.5 Statistical analyses

We calculated principal components for the recorded courtship behaviours and for parental care behaviour (Tables S3 and S4). To assess if activity changed from the first to the second cycle depending on treatment, we used mixed models with treatment and cycle as fixed factors and male as random factor. To investigate the pattern in more detail, we separately tested if temperature influenced activity during the first cycle, using linear models with temperature as fixed factor, and, further, if activity changed from the first to the second cycle within each treatment, using mixed models with cycle as fixed factor and male as random factor. To consider the multiple use of some females, female identity was inserted into the models as a random factor. No significant effect of female identity was detected and we removed the factor from the models, which did not influence the results. All analyses were performed using the software IBM spss Statistics 26. We checked that the assumptions of the tests regarding heteroscedasticity and normal distribution of residuals were held.

## 3 RESULTS

**FIGURE 1:**
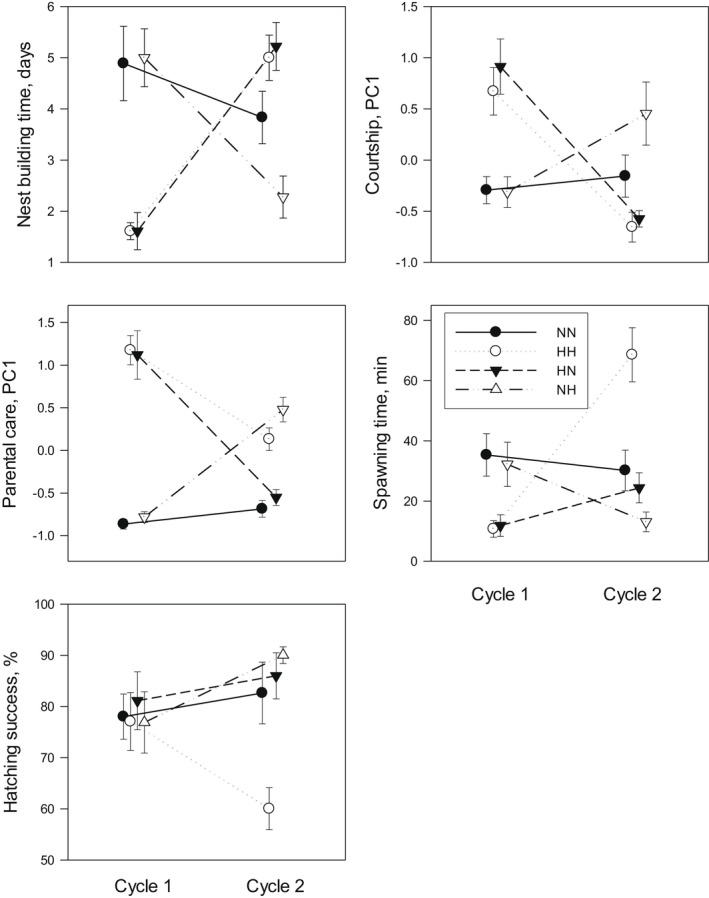
Reproductive behaviours and hatching success of threespine stickleback under different temperature conditions during a first and a second breeding cycle. Data shows mean ± *SE*, *N* = 18. NN: normal temperature during both cycles; HH: high temperature during both cycles; HN: high temperature during first cycle; NH: high temperature during second cycle.

Changes in activity from the first to the second cycle depended on treatment, as indicated by significant interactions between treatment and cycle for the recorded activities (Table 1). During the first breeding cycle, males reproducing at the higher temperature (19°C) were more active than males reproducing at the normal temperature (14°C) for the recorded behaviours: nest building (*F*_1,70_ = 45.39, *p* < .001), courtship (*F*_1,70_ = 23.78, *p* < .001), and parental care (*F*_1,70_ = 135.49, *p* < .001). Males built a nest faster, courted more vigorously, and spent more time on parental care (Figure 1). During the second breeding cycle, males at constant normal temperature (NN) did not alter their behaviour from the first cycle, while males at constant high temperature (HH) took longer to build a nest and reduced their courtship and parental care activity (Table 2). Males exposed to high temperature during the first cycle but not the second (HN) were slower at building a nest and reduced their courtship and parental care activity, while males exposed to high temperature during the second but not the first cycle (NH) showed the opposite pattern with faster nest building and increased courtship and parental care activity (Table 2). Thus, parental effort was temperature dependent except for males at constant high temperature (HH), who were not able to maintain their high activity during the second cycle.

**TABLE 1:** Influence of treatment (NN, HH, HN and NH, see text for explanation) and breeding cycle (first or second) on reproductive behaviours and hatching success of threespine stickleback males.

	Treatment		Cycle		Interaction	
	*F*	*p*	*F*	*p*	*F*	*p*
Nest building time	1.94	.126	5.60	.019	21.89	<.001
Courtship (PC1)	1.08	.365	13.39	<.001	19.76	<.001
Parental care (PC1)	27.71	<.001	12.25	.001	51.088	<.001
Spawning time	4.11	.010	10.44	.002	22.08	<.001
Hatching success	3.84	.013	0.18	.672	3.82	.014

*Note*: *N* = 18 for each of the four treatments. Mixed models were used to analyse the data with treatment and cycle as fixed factors and male as random factor.

**TABLE 2:** Changes in reproductive behaviours and hatching success from the first to the second breeding cycle for threespine stickleback males in the four treatments: constant normal temperature (NN), constant high temperature (HH), high temperature during the first but not the second cycle (HN) and high temperature during the second but not the first cycle (NH).

	NN		HH		HN		NH	
	*F*	*p*	*F*	*p*	*F*	*p*	*F*	*p*
Nest building time	1.41	.244	51.47	<.001	39.01	<.001	18.34	.001
Courtship (PC1)	0.74	.403	30.32	<.001	32.96	<.001	6.50	.021
Parental care	3.33	.086	70.30	<.001	31.26	<.001	107.25	<.001
Spawning time	0.42	.520	48.89	<.001	5.39	.033	8.66	.009
Hatching success	0.39	.540	6.93	.017	0.94	.350	4.52	.041

*Note*: *N* = 18 per treatment. Mixed models were used to analyse the data, with cycle as fixed factor and male as random factor.

Females spawned sooner during the first breeding cycle when temperature was high (*F*_1,70_ = 16.70, *p* < .001). During the second cycle, time until spawning did not differ from the first cycle at constant normal temperature (NN) but was extended at constant high temperature (HH) and when temperature dropped (HN), while the time was shortened when temperature rose (NH) (Table 2). Thus, time until spawning was temperature dependent except during the second cycle at constant high temperature when males were less successful in attracting females.

The eggs hatched earlier at the higher temperature during both the first (*F*_1,70_ = 1200.71, *p* < .001) and the second cycle (*F*_1,70_ = 1735.48, *p* < .001). Hatching success (percent of eggs hatching) did not depend on temperature during the first cycle (*F*_1,70_ = 0.09, *p* = .76). During the second cycle, males at constant high temperature (HH) had a lower hatching success than during the first cycle, while males at constant normal temperature (NN) and males experiencing a fall in temperature (HN) showed no change in hatching success (Table 2). Males experiencing a rise in temperature (NH) showed a slight improvement in hatching success (Table 2).

Males completed the two breeding cycles faster when maintained at constant high temperature (HH) (18 days ± 1, mean ± *SE*) than at constant normal temperature (NN) (33 days ± 1, *F*_1,34_ = 122.61, *p* < .001), falling temperature (HN) (26 days ± 1, *F*_1,34_ = 42.66, *p* < .001) or rising temperature (NH) (26 days ± 1, *F*_1,34_ = 52.04, *p* < .001, see Table S2 for duration of each cycle). Males at constant high temperature (HH) lost more body weight (28% ± 1) than males at constant normal temperature (NN) (9% ± 1, *F*_1,34_ = 145.95, *p* < .001), falling temperature (HN) (21% ± 1, *F*_1,34_ = 12.23, *p* = .001), or rising temperature (NH) (21% ± 2, *F*_1,34_ = 18.09, *p* < .001). Males experiencing a rise or fall in temperature (NH or HN) did not differ in the time it took to complete the two breeding cycles (*F*_1,34_ = 0.01, *p* = .91) or in weight loss (*F*_34_ = 0.01, *p* = .93).

## 4 DISCUSSION

Our results show that threespine stickleback males adjust their reproductive behaviours—nest building, courtship and parental care—to changes in water temperature. They increase their activity when temperature rises and decrease it when temperature drops, as expected for ectothermic organisms (Dillon et al., 2010; Sinclair et al., 2016). However, when temperature continues to be high over a longer period—across two breeding cycles—males fail to maintain their high activity during the second cycle, which reduces their ability to attract females to spawn and to raise eggs in the nest to the hatching stage. Males that experience high temperature only during one breeding cycle (first or second), and who increase their activity only during this short period, are, on the other hand, able to maintain high reproductive success across the two breeding cycles. Thus, enhanced reproductive activity during a short spell of high temperature is adaptive but not sustainable over longer periods. Our expectation of sustained periods of higher temperature being more stressful than short periods was consequently upheld. These results show that a species that has been exposed to temperature fluctuations in its breeding habitat in the past, and is expected to withstand heatwaves better than many other species, fails to adjust to prolonged periods of high temperature and suffers a reduction in offspring production.

During the short spell of high temperature, males increased their reproductive activities irrespective of whether the spell occurred early or late during the breeding season. Thus, our expectation of males being less able to adjust to temperature increases later in the season, due to a gradual decrease in body condition (Candolin, 2000a), was not upheld. Given that higher temperatures are more common later in the breeding season, selection for adjusting to higher temperatures at this time of the year, also when condition starts to deteriorate, has probably been strong. Whether an effect of temperature would have emerged during a third cycle, as some males are able to complete three breeding cycles (Candolin, 2000a), remains to be determined.

The increased activity during a short spell of higher temperature was most likely a consequence of faster metabolic rate, as is generally the case in ectotherms (Dillon et al., 2010; Sinclair et al., 2016). In addition, changes in female activity could have contributed to the enhanced courtship activity, as female metabolic rate also increases with temperature. Moreover, females could have been more willing to spawn at the higher temperature, as increased temperature accelerates the maturation of eggs and shortens the time window available for females to search for breeding males (Pankhurst & Munday, 2011). Regarding the recorded increase in male parental care activity, this is required for successful rearing of eggs, as higher temperature lowers water oxygen saturation and accelerates the metabolic rate of eggs (Pankhurst & Munday, 2011; Smyder & Martin, 2002). Thus, the increased parental care activity during a short spell of higher temperature was adaptive, while the failure of males to maintain the high parental care activity during a longer period of high temperature apparently contributed to their lower hatching success.

The cause of the inability of males to maintain high reproductive activity under sustained high temperature—during the second breeding cycle—was probably exhaustion. This is supported by males losing more weight in the sustained high temperature treatment than in the other treatments. In addition, the nest building period between the two breeding cycles was prolonged for males on high temperature during the first cycle, which indicates the need for a longer recovery time. This prolongation partly cancelled out the benefit of a shorter parental care period, which otherwise could increase the number of breeding cycles that males could complete (Candolin, 2000a). Changes in female behaviour cannot explain the reduction in male activity and reproductive success during the second cycle (for males on sustained high temperature), as males exposed to high temperature only during the second cycle did not show a decline in activity or reproductive success (the females had experienced the same conditions as females in the sustained high temperature treatment). Similarly, a lower quality of eggs or fertilisation success cannot explain the lower hatching success of males on sustained high temperature (Mehlis & Bakker, 2014), as lower success was not recorded for males exposed to high temperature only during the second cycle.

These results indicate that threespine stickleback males are able to cope with shorter spells of higher temperature, but not when their duration is prolonged. Yet, such extreme conditions are becoming increasingly common. The investigated stickleback population has apparently adapted to short periods of high temperature, as these have occurred also in the past, but not to their prolongation. Thus, responses to short‐term extreme conditions cannot be used to predict responses to longer‐term changes for the investigated species. This stresses the importance of considering the duration of extreme conditions, such as heatwaves, when evaluating the ability of organisms to cope with the ongoing climate change. Investigations often focus on the limits of temperature tolerance, while effects of the duration of high temperature is seldom considered (Lawson et al., 2015; Rezende et al., 2014; Sinclair et al., 2016).

The results further stress the importance of considering the past history of species when predicting and assessing responses to extreme conditions (Chevin & Hoffmann, 2017; Sih et al., 2011; Tuomainen & Candolin, 2011). Although the experimental conditions only extended natural conditions, the population was unable to cope with their prolongation. The impact of prolonged periods of higher temperature could be even worse for species originating from more stable environments. Yet, little is currently known about the impact that past conditions have on the ability of species to cope with heatwaves (Bay et al., 2017; Candolin & Jensen, 2021; Lasky et al., 2020; Lavergne et al., 2013; Lawson et al., 2015; McGaughran et al., 2021).

To conclude, our results show that threespine stickleback males are able to adaptively adjust their reproductive behaviours to short‐term increases in temperature, but that the prolonged duration of high temperature poses a challenge that reduces the activity and reproductive success of stickleback. Thus, the average reaction norm that has evolved in the population is insufficient for coping with temperature rises when the duration of high temperature is prolonged. These results emphasise the importance of considering the duration of extreme conditions when assessing the impact of climate change on species. Responses to short‐term exposures cannot be extrapolated to assess responses to sustained periods of extreme conditions.

## CONFLICT OF INTEREST

The authors declare no conflict of interest.

## Supporting information


Appendix S1

## Data Availability

The data that support the findings of this study are available in the Dryad Digital Repository (https://doi.org/10.5061/dryad.nzs7h44tt) (Isotalo et al., 2022).

## References

[gcb16381-bib-0001] Abram, P. K. , Boivin, G. , Moiroux, J. , & Brodeur, J. (2017). Behavioural effects of temperature on ectothermic animals: Unifying thermal physiology and behavioural plasticity. Biological Reviews, 92(4), 1859–1876. 10.1111/brv.12312 28980433

[gcb16381-bib-0002] Angiletta, M. J. (2009). Thermal adaptation: A theoretical and empirical synthesis. Oxford University Press.

[gcb16381-bib-0003] Antao, L. H. , Bates, A. E. , Blowes, S. A. , Waldock, C. , Supp, S. R. , Magurran, A. E. , Dornelas, M. , & Schipper, A. M. (2020). Temperature‐related biodiversity change across temperate marine and terrestrial systems. Nature Ecology and Evolution, 4(7), 927–933. 10.1038/s41559-020-1185-7 32367031

[gcb16381-bib-0004] Barrett, R. D. H. , & Hendry, A. P. (2012). Evolutionary rescue under environmental change? In U. Candolin & B. B. M. Wong (Eds.), Behavioural responses to a changing world. Mechanisms and consequences (pp. 216–233). Oxford University Press.

[gcb16381-bib-0005] Bay, R. A. , Rose, N. , Barrett, R. , Bernatchez, L. , Ghalambor, C. K. , Lasky, J. R. , Brem, R. B. , Palumbi, S. R. , & Ralph, P. (2017). Predicting responses to contemporary environmental change using evolutionary response architectures. American Naturalist, 189(5), 463–473. 10.1086/691233 28410032

[gcb16381-bib-0006] Bernal, M. A. , Schunter, C. , Lehmann, R. , Lightfoot, D. J. , Allan, B. J. M. , Veilleux, H. D. , Rummer, J. L. , Munday, P. L. , & Ravasi, T. (2020). Species‐specific molecular responses of wild coral reef fishes during a marine heatwave. Science Advances, 6(12), eaay3423. 10.1126/sciadv.aay3423 32206711 PMC7080449

[gcb16381-bib-0007] Brandt, E. E. , Kelley, J. P. , & Elias, D. O. (2018). Temperature alters multimodal signaling and mating success in an ectotherm. Behavioral Ecology and Sociobiology, 72(12), 14. 10.1007/s00265-018-2620-5

[gcb16381-bib-0008] Candolin, U. (1997). Predation risk affects courtship and attractiveness of competing threespine stickleback males. Behavioral Ecology and Sociobiology, 41, 81–87.

[gcb16381-bib-0009] Candolin, U. (2000a). Changes in expression and honesty of sexual signalling over the reproductive lifetime of sticklebacks. Proceedings of the Royal Society B: Biological Sciences, 267, 2425–2430. 10.1098/rspb.2000.1301 PMC1690825 11133033

[gcb16381-bib-0010] Candolin, U. (2000b). Increased signalling effort when survival prospects decrease: Male–male competition ensures honesty. Animal Behaviour, 60, 417–422. 11032643 10.1006/anbe.2000.1481

[gcb16381-bib-0011] Candolin, U. (2000c). Male‐male competition ensures honest signaling of male parental ability in the three‐spined stickleback. Behavioral Ecology and Sociobiology, 49, 57–61.

[gcb16381-bib-0012] Candolin, U. (2019). Mate choice in a changing world. Biological Reviews, 94(4), 1246–1260. 10.1111/brv.12501 30762277

[gcb16381-bib-0013] Candolin, U. , Goncalves, S. , & Pant, P. (2021). Parental care amplifies changes in offspring production in a disturbed environment. Oikos, 130(12), 2231–2238. 10.1111/oik.08668

[gcb16381-bib-0014] Candolin, U. , Goncalves, S. , & Pant, P. (2022). Delayed early life effects in the threespine stickleback. Proceedings of the Royal Society B: Biological Sciences, 289, 20220554. 10.1098/rspb.2022.0554 PMC9156908 35642365

[gcb16381-bib-0015] Candolin, U. , & Jensen, I. (2021). Phenotypic plasticity in courtship exposed to selection in a human‐disturbed environment. Evolutionary Applications., 14, 2392–2401. 10.1111/eva.13225 34745333 PMC8549619

[gcb16381-bib-0016] Candolin, U. , & Salesto, T. (2006). Effects of increased vegetation cover on nesting behavior of sticklebacks (*Gasterosteus aculeatus*). Behavioral Ecology and Sociobiology, 59(5), 689–693. 10.1007/s00265-005-0098-4

[gcb16381-bib-0017] Candolin, U. , & Voigt, H. R. (2001). No effect of a parasite on reproduction in stickleback males: A laboratory artefact? Parasitology, 122, 457–464. 10.1017/S0031182001007600 11315179

[gcb16381-bib-0018] Chevin, L. M. (2013). Genetic constraints on adaptation to a changing environment. Evolution, 67(3), 708–721. 10.1111/j.1558-5646.2012.01809.x 23461322

[gcb16381-bib-0019] Chevin, L. M. , & Hoffmann, A. A. (2017). Evolution of phenotypic plasticity in extreme environments. Philosophical Transactions of the Royal Society B: Biological Sciences, 372(1723), 12. 10.1098/rstb.2016.0138 PMC5434089 28483868

[gcb16381-bib-0020] Chevin, L. M. , Lande, R. , & Mace, G. M. (2010). Adaptation, plasticity, and extinction in a changing environment: Towards a predictive theory. PLoS Biology, 8(4), e1000357. 10.1371/journal.pbio.1000357 20463950 PMC2864732

[gcb16381-bib-0021] Conrad, T. , Stocker, C. , & Ayasse, M. (2017). The effect of temperature on male mating signals and female choice in the red mason bee, *Osmia bicornis* (L.). Ecology and Evolution, 7(21), 8966–8975. 10.1002/ece3.3331 29152191 PMC5677480

[gcb16381-bib-0022] Dell, A. I. , Pawar, S. , & Savage, V. (2014). Temperature dependence of trophic interactions are driven by asymmetry of species responses and foraging strategy. Journal of Animal Ecology, 83(1), 70–84. 10.1111/1365-2656.12081 23692182

[gcb16381-bib-0023] Dillon, M. E. , Wang, G. , & Huey, R. B. (2010). Global metabolic impacts of recent climate warming. Nature, 467(7316), 704–706. 10.1038/nature09407 20930843

[gcb16381-bib-0024] Fox, R. J. , Donelson, J. M. , Schunter, C. , Ravasi, T. , & Gaitan‐Espitia, J. D. (2019). Beyond buying time: The role of plasticity in phenotypic adaptation to rapid environmental change. Philosophical Transactions of the Royal Society B: Biological Sciences, 374(1768), 20180174. 10.1098/rstb.2018.0174 PMC6365870 30966962

[gcb16381-bib-0025] Friard, O. , & Gamba, M. (2016). BORIS: A free, versatile open‐source event‐logging software for video/audio coding and live observations. Methods in Ecology and Evolution, 7(11), 1325–1330. 10.1111/2041-210x.12584

[gcb16381-bib-0026] Fuxjager, L. , Wanzenbock, S. , Ringler, E. , Wegner, K. M. , Ahnelt, H. , & Shama, L. N. S. (2019). Within‐generation and transgenerational plasticity of mate choice in oceanic stickleback under climate change. Philosophical Transactions of the Royal Society B: Biological Sciences, 374(1768), 20180183. 10.1098/rstb.2018.0183 PMC6365864 30966960

[gcb16381-bib-0027] Granroth‐Wilding, H. M. V. , & Candolin, U. (2022). No strong associations between temperature and the host–parasite interaction in wild stickleback. Journal of Fish Biology (in press). 10.1111/jfb.15107 PMC9545309 35598110

[gcb16381-bib-0028] Harvey, B. , Marshall, K. E. , Harley, C. D. G. , & Russell, B. D. (2022). Predicting responses to marine heatwaves using functional traits. Trends in Ecology & Evolution, 37(1), 20–29. 10.1016/j.tree.2021.09.003 34593256

[gcb16381-bib-0029] Hedrick, A. V. , Perez, D. , Lichti, N. , & Yew, J. (2002). Temperature preferences of male field crickets (*Gryllus integer*) alter their mating calls. Journal of Comparative Physiology A: Neuroethology Sensory Neural and Behavioral Physiology, 188(10), 799–805. 10.1007/s00359-002-0368-9 12466955

[gcb16381-bib-0030] Hendry, A. P. , Farrugia, T. J. , & Kinnison, M. T. (2008). Human influences on rates of phenotypic change in wild animal populations. Molecular Ecology, 17(1), 20–29. 18173498 10.1111/j.1365-294X.2007.03428.x

[gcb16381-bib-0031] Isotalo, T. , Rotenbiller, L. , & Candolin, U. (2022). The importance of considering the duration of extreme temperatures when investigating responses to climate change. *Dryad Digital Repository* . 10.5061/dryad.nzs7h44tt PMC9805119 36053986

[gcb16381-bib-0032] La Sorte, F. A. , Johnston, A. , & Ault, T. R. (2021). Global trends in the frequency and duration of temperature extremes. Climatic Change, 166, 1. 10.1007/s10584-021-03094-0

[gcb16381-bib-0033] Lasky, J. R. , Hooten, M. B. , & Adler, P. B. (2020). What processes must we understand to forecast regional‐scale population dynamics? Proceedings of the Royal Society B: Biological Sciences, 287(1940), 20202219. 10.1098/rspb.2020.2219 PMC7739927 33290672

[gcb16381-bib-0034] Lavergne, S. , Evans, M. E. K. , Burfield, I. J. , Jiguet, F. , & Thuiller, W. (2013). Are species' responses to global change predicted by past niche evolution? Philosophical Transactions of the Royal Society B: Biological Sciences, 368(1610), 20120091. 10.1098/rstb.2012.0091 PMC3538457 23209172

[gcb16381-bib-0035] Lawson, C. R. , Vindenes, Y. , Bailey, L. , & van de Pol, M. (2015). Environmental variation and population responses to global change. Ecology Letters, 18(7), 724–736. 10.1111/ele.12437 25900148

[gcb16381-bib-0036] Le Cren, E. D. (1951). The length–weight relationship and seasonal cycle in gonad weight and condition in the perch (*Perca fluviatilis*). Journal of Animal Ecology, 20(2), 201–219. 10.2307/1540

[gcb16381-bib-0037] Macchiano, A. , Sasson, D. A. , Leith, N. T. , & Fowler‐Finn, K. D. (2019). Patterns of thermal sensitivity and sex‐specificity of courtship behavior differs between two sympatric species of *Enchenopa* treehopper. Frontiers in Ecology and Evolution, 7, 11. 10.3389/fevo.2019.00361

[gcb16381-bib-0038] McGaughran, A. , Laver, R. , & Fraser, C. (2021). Evolutionary responses to warming. Trends in Ecology & Evolution, 36(7), 591–600. 10.1016/j.tree.2021.02.014 33726946

[gcb16381-bib-0039] McLean, N. , Lawson, C. R. , Leech, D. I. , & van de Pol, M. (2016). Predicting when climate‐driven phenotypic change affects population dynamics. Ecology Letters, 19(6), 595–608. 10.1111/ele.12599 27062059

[gcb16381-bib-0040] Mehlis, M. , & Bakker, T. C. M. (2014). The influence of ambient water temperature on sperm performance and fertilization success in three‐spined sticklebacks (*Gasterosteus aculeatus*). Evolutionary Ecology, 28(4), 655–667. 10.1007/s10682-014-9707-x

[gcb16381-bib-0041] Meier, H. E. M. , Dieterich, C. , Eilola, K. , Gröger, M. , Höglund, A. , Radtke, H. , Saraiva, S. , & Wåhlström, I. (2019). Future projections of record‐breaking sea surface temperature and cyanobacteria bloom events in the Baltic Sea. Ambio, 48(11), 1362–1376. 10.1007/s13280-019-01235-5 31506843 PMC6814679

[gcb16381-bib-0042] Meier, H. E. M. , Kniebusch, M. , Dieterich, C. , Groger, M. , Zorita, E. , Elmgren, R. , Myrberg, K. , Ahola, M. P. , Bartosova, A. , Bonsdorff, E. , Börgel, F. , Capell, R. , Carlén, I. , Carlund, T. , Carstensen, J. , Christensen, O. B. , Dierschke, V. , Frauen, C. , Frederiksen, M. , … Zhang, W. Y. (2022). Climate change in the Baltic Sea region: A summary. Earth System Dynamics, 13(1), 457–593. 10.5194/esd-13-457-2022

[gcb16381-bib-0043] Merila, J. , & Hendry, A. P. (2014). Climate change, adaptation, and phenotypic plasticity: The problem and the evidence. Evolutionary Applications, 7(1), 1–14. 10.1111/eva.12137 24454544 PMC3894893

[gcb16381-bib-0044] Nissling, A. , Nyberg, S. , & Petereit, C. (2017). Egg buoyancy of flounder, *Platichthys flesus*, in the Baltic Sea‐adaptation to salinity and implications for egg survival. Fisheries Research, 191, 179–189. 10.1016/j.fishres.2017.02.020

[gcb16381-bib-0045] Noer, N. K. , Orsted, M. , Schiffer, M. , Hoffmann, A. A. , Bahrndorff, S. , & Kristensen, T. N. (2022). Into the wild‐a field study on the evolutionary and ecological importance of thermal plasticity in ectotherms across temperate and tropical regions. Philosophical Transactions of the Royal Society B: Biological Sciences, 377(1846), 20210004. 10.1098/rstb.2021.0004 PMC8784925 35067088

[gcb16381-bib-0046] Oliver, E. C. J. , Benthuysen, J. A. , Darmaraki, S. , Donat, M. G. , Hobday, A. J. , Holbrook, N. J. , Schlegel, R. W. , & Sen Gupta, A. (2021). Marine heatwaves. Annual Review of Marine Science, 13, 313–342. 10.1146/annurev-marine-032720-095144 32976730

[gcb16381-bib-0047] Pankhurst, N. W. , & Munday, P. L. (2011). Effects of climate change on fish reproduction and early life history stages. Marine and Freshwater Research, 62(9), 1015–1026. 10.1071/mf10269

[gcb16381-bib-0048] Rezende, E. L. , Castaneda, L. E. , & Santos, M. (2014). Tolerance landscapes in thermal ecology. Functional Ecology, 28(4), 799–809. 10.1111/1365-2435.12268

[gcb16381-bib-0049] Roman‐Palacios, C. , & Wiens, J. J. (2020). Recent responses to climate change reveal the drivers of species extinction and survival. Proceedings of the National Academy of Sciences of the United States of America, 117(8), 4211–4217. 10.1073/pnas.1913007117 32041877 PMC7049143

[gcb16381-bib-0050] Rosenthal, M. F. , & Elias, D. O. (2019). Nonlinear changes in selection on a mating display across a continuous thermal gradient. Proceedings of the Royal Society B: Biological Sciences, 286(1907), 7. 10.1098/rspb.2019.1450 PMC6661355 31337317

[gcb16381-bib-0051] Sih, A. , Ferrari, M. C. O. , & Harris, D. J. (2011). Evolution and behavioural responses to human‐induced rapid environmental change. Evolutionary Applications, 4(2), 367–387. 10.1111/j.1752-4571.2010.00166.x 25567979 PMC3352552

[gcb16381-bib-0052] Sinclair, B. J. , Marshall, K. E. , Sewell, M. A. , Levesque, D. L. , Willett, C. S. , Slotsbo, S. , Dong, Y. , Harley, C. D. , Marshall, D. J. , Helmuth, B. S. , & Huey, R. B. (2016). Can we predict ectotherm responses to climate change using thermal performance curves and body temperatures? Ecology Letters, 19(11), 1372–1385. 10.1111/ele.12686 27667778

[gcb16381-bib-0053] Smale, D. A. , Wernberg, T. , Oliver, E. C. J. , Thomsen, M. , Harvey, B. P. , Straub, S. C. , Burrows, M. T. , Alexander, L. V. , Benthuysen, J. A. , Donat, M. G. , Feng, M. , Hobday, A. J. , Holbrook, N. J. , Perkins‐Kirkpatrick, S. E. , Scannell, H. A. , Gupta, A. S. , Payne, B. L. , & Moore, P. J. (2019). Marine heatwaves threaten global biodiversity and the provision of ecosystem services. Nature Climate Change, 9(4), 306–312. 10.1038/s41558-019-0412-1

[gcb16381-bib-0054] Smyder, E. A. , & Martin, K. L. M. (2002). Temperature effects on egg survival and hatching during the extended incubation period of California grunion, *Leuresthes tenuis* . Copei, 2, 313–320.

[gcb16381-bib-0055] Spinks, R. K. , Bonzi, L. C. , Ravasi, T. , Munday, P. L. , & Donelson, J. M. (2021). Sex‐ and time‐specific parental effects of warming on reproduction and offspring quality in a coral reef fish. Evolutionary Applications, 14(4), 1145–1158. 10.1111/eva.13187 33897826 PMC8061261

[gcb16381-bib-0056] Stillman, J. H. (2019). Heat waves, the new Normal: Summertime temperature extremes will impact animals, ecosystems, and human communities. Physiology, 34(2), 86–100. 10.1152/physiol.00040.2018 30724132

[gcb16381-bib-0057] Suryan, R. M. , Arimitsu, M. L. , Coletti, H. A. , Hopcroft, R. R. , Lindeberg, M. R. , Barbeaux, S. J. , Batten, S. D. , Burt, W. J. , Bishop, M. A. , Bodkin, J. L. , Brenner, R. , Campbell, R. W. , Cushing, D. A. , Danielson, S. L. , Dorn, M. W. , Drummond, B. , Esler, D. , Gelatt, T. , Hanselman, D. H. , … Zador, S. G. (2021). Ecosystem response persists after a prolonged marine heatwave. Scientific Reports, 11(1), 6235. 10.1038/s41598-021-83818-5 33737519 PMC7973763

[gcb16381-bib-0058] Tuomainen, U. , & Candolin, U. (2011). Behavioural responses to human‐induced environmental change. Biological Reviews, 86(3), 640–657. 10.1111/j.1469-185X.2010.00164.x 20977599

[gcb16381-bib-0059] Tuomainen, U. , & Candolin, U. (2013). Environmental change and extended phenotypes: Does eutrophication influence nest building in sticklebacks? Ethology, 119(6), 503–510. 10.1111/eth.12088

[gcb16381-bib-0060] Urban, M. C. , Bocedi, G. , Hendry, A. P. , Mihoub, J. B. , Pe'er, G. , Singer, A. , Bridle, J. R. , Crozier, L. G. , de Meester, L. , Godsoe, W. , Gonzalez, A. , Hellmann, J. J. , Holt, R. D. , Huth, A. , Johst, K. , Krug, C. B. , Leadley, P. W. , Palmer, S. C. , Pantel, J. H. , … Travis, J. M. (2016). Improving the forecast for biodiversity under climate change. Science, 353(6304), 1113. 10.1126/science.aad8466 27609898

[gcb16381-bib-0061] Vinagre, C. , Mendonca, V. , Cereja, R. , Abreu‐Afonso, F. , Dias, M. , Mizrahi, D. , & Flores, A. A. V. (2018). Ecological traps in shallow coastal waters‐potential effect of heat‐waves in tropical and temperate organisms. PLoS One, 13(2), 17. 10.1371/journal.pone.0192700 PMC5805332 29420657

[gcb16381-bib-0062] Wanzenboeck, S. , Fuxjaeger, L. , Ringler, E. , Ahnelt, H. , & Shama, L. N. S. (2022). Temperature‐dependent reproductive success of stickleback lateral plate morphs: Implications for population polymorphism and range shifts under ocean warming. Frontiers in Marine Science, 9, 759450. 10.3389/fmars.2022.759450

