# Supplementary material for: The importance of considering the duration of extreme temperatures when investigating responses to climate change
Source: Glob Chang Biol. 2022 Aug 19;28(22):6577–85. doi: 10.1111/gcb.16381 (PMC9805119; doi:10.1111/gcb.16381)
Supplement: Supplementary file 1 — Appendix S1 [file GCB-28-6577-s001.pdf]

Supporting Information for

Duration of temperature extremes determines coping ability in the threespine stickleback

Teija Isotalo, Lilla Rotenbiller and Ulrika Candolin

Map of the location of the spawning habitat of the investigated threespine stickleback populations

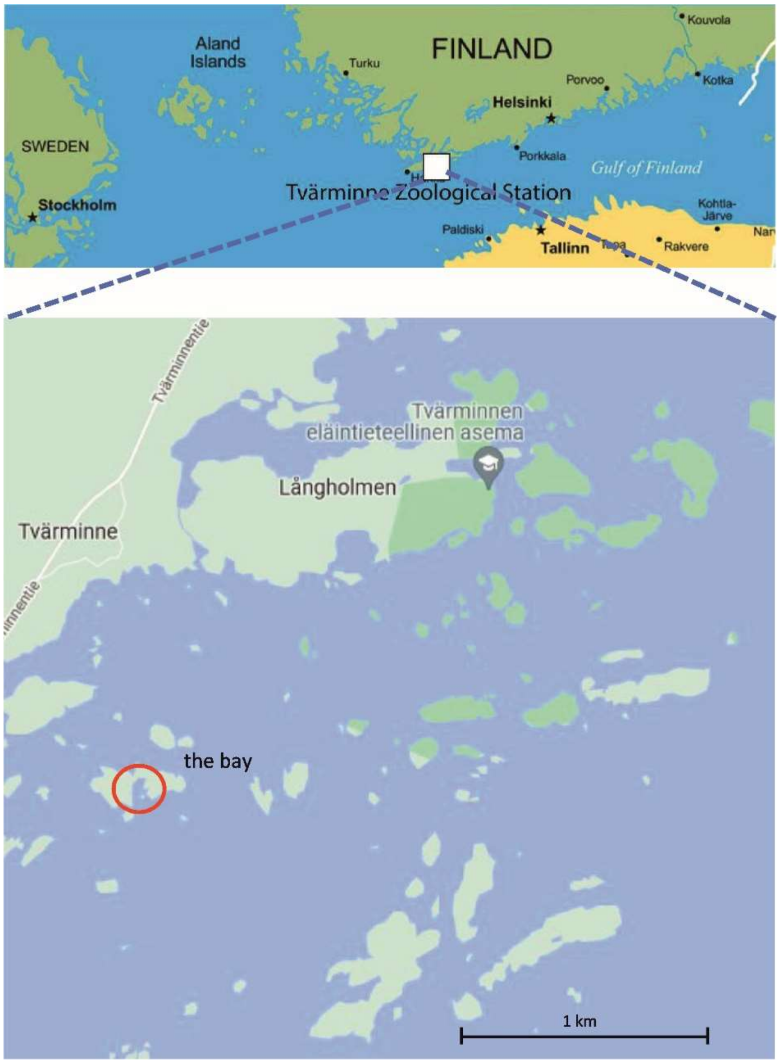

Table S1 Temperature in the spawning habitat of the investigated population in June during the spawning period.

Year	Temperature °C	
	Mean	Variance
2010	14.1	2.1
2011	13.4	1.5
2012	12.9	1.1
2013	14.8	2.0
2014	14.6	1.8
2015	13.9	1.4
2016	14.5	1.3
2017	13.3	1.1
2018	15.0	1.5

Table S2. Male standard length and weight, and the duration of the two breeding cycles, in the four treaments: NN, NH, HN, and HH (N = normal temperature, H = high temperature, during first and second breeding cycle).

Treatment		Male standard length (mm)	Male wet weight (g)	Duration of breeding cycles (days)	
				Cycle 1	Cycle 2
**NN**	Mean	61	2.22	18.3	15.0
	N	18	18	18	18
	SD	4	0.40	31	2.7
**NH**	Mean	61	2.17	18,6	7.7
	N	18	18	18	18
	SD	4	0.39	2.7	1.5
**HN**	Mean	63	2.49	9.8	16.3
	N	18	18	18	18
	SD	4	0.55	2.6	3.9
**HH**	Mean	62	2.34	8.4	10.3
	N	18	18	18	18
	SD	4	0.47	1.0	3.8

Table S3. Principal components for courtship behaviours

Total Variance Explained

	Initial Eigenvalues			Extraction Sums of Squared Loadings		
Component	Total	% of Variance	Cumulative %	Total	% of Variance	Cumulative %
1	2,905	72,613	72,613	2,905	72,613	72,613
2	,728	18,192	90,805			
3	,296	7,393	98,198			
4	,072	1,802	100,000			

Component Matrix

	Component 1
Mean leads	,896
Mean fanning bouts	,948
Mean fanning time	,914
Mean courting time	,607

Table S4. Prinipal components for parental care behaviours

Total Variance Explained

	Initial Eigenvalues			Extraction Sums of Squared Loadings		
Component	Total	% of Variance	Cumulative %	Total	% of Variance	Cumulative %
1	2,839	94,633	94,633	2,839	94,633	94,633
2	,133	4,428	99,061			
3	,028	,939	100,000			

Component Matrix

	Component 1
Fanning bouts	,954
Fanning time	,983
Nest directed behaviours, time	,981
